# Congenital infiltrative lipomas and retroperitoneal perirenal lipomas in a calf

**DOI:** 10.1186/s13028-016-0200-0

**Published:** 2016-03-05

**Authors:** Jørgen S. Agerholm, Fintan J. McEvoy, Michael H. Goldschmidt

**Affiliations:** 1 Department of Large Animal Sciences, Faculty of Health and Medical Sciences, University of Copenhagen, Dyrlægevej 68, 1870 Frederiksberg C, Denmark; 2 Department of Veterinary Clinical and Animal Sciences, Faculty of Health and Medical Sciences, University of Copenhagen, Dyrlægevej 16, 1870 Frederiksberg C, Denmark; 3 Department of Pathobiology, School of Veterinary Medicine, University of Pennsylvania, Philadelphia, PA 19104-6051 USA

**Keywords:** Bovine, Neoplasia, Malformation, Defect

## Abstract

**Background:**

Congenital lipocytic tumours have rarely been reported in cattle. Lipomas are benign tumours, but infiltrative lipomas have significant health implications due to their aggressive infiltrative growth pattern.

**Case presentation:**

A calf was born with skeletal malformations and soft tissue proliferations, primarily on the external thoracic wall. The calf was euthanized for welfare reasons and submitted for post mortem examination. Necropsy, histopathology and post mortem computed tomography scanning revealed two types of lipocytic tumours. Widespread infiltrative lipomas were present in the muscles and connective tissues along the vertebral column and diffusely invaded the external soft tissues of the right thoracic wall. The neoplastic lipocytes had invaded intervertebral spaces thus causing congenital vertebral malformations, and further invaded the vertebral canal and the bone marrow of coccygeal vertebrae. Periosteal localization of the tumour was associated with costal hyperostosis. Two large retroperitoneal lipomas enclosed the kidneys and occupied much of the abdominal space.

**Conclusion:**

The development of congenital bone malformation in this calf illustrates the severe consequences of the infiltrative and aggressive growth of infiltrative lipomas during foetal development. The congenital retroperitoneal lipomas occupied a large part of abdominal cavity, but did not invade the adjacent tissues. Due to their large size, perirenal lipomas should be considered in calves with distended abdomen, even in cases without other signs of tumours.

## Background

Lipocytic tumours in domestic animals are usually found in adult to aged individuals, while congenital forms have been reported infrequently. Lipocytic tumours are grouped into pure lipocytic tumours, i.e., lipomas, infiltrative lipomas and liposarcomas [1] and mixed cell types such as fibrolipomas. Congenital lipocytic tumours in cattle seem to be rare and only a few cases have been reported. Congenital infiltrative lipoma, which is a benign but locally invasive tumour composed of well-differentiated lipocytes, has been reported twice [2, 3]. Other reports of congenital lipocytic tumours include an intracranial lipoma that expanded into the subcutaneous tissue [4], a subcutaneous fibrolipoma [5], bilateral retroperitoneal perirenal lipomas [6] and a tumour diagnosed as a lipoblastoma at the base of the tail [7]. Due to the limited knowledge of congenital tumours in cattle, further reports on such conditions are needed. Here, we report a calf having both widespread congenital infiltrative lipomas associated with skeletal malformation and invasion of bone marrow, and bilateral retroperitoneal perirenal lipomas.

## Case presentation

A female Danish Red dairy calf (no. 1) was delivered on gestation day 270 after an uncomplicated calving as twin to a normally developed female calf (no. 2). The offspring were severely inbred as the pregnancy was the result of unintended insemination with semen of the dam’s own sire. The cow had previously given birth to three normal calves and was clinically normal. Calf 1 was euthanized by the use of a captive bolt pistol and exsanguination immediately after birth for welfare reasons due to skeletal malformations. Calf 2 had reduced growth rate and was acquired at the age of 50 days, transported to the University of Copenhagen and euthanized by intravenous injection of an overdose of pentobarbital sodium.

The calves were submitted for further examination as part of the Danish Bovine Genetic Disease Programme [8] due to suspicion of a genetic syndrome expressed because of the inbreeding. They originated from a herd free of bovine virus diarrhea virus infection and a number of other infections officially eradicated from Denmark [9]. The calves were necropsied, but the spine of case 1 also underwent post mortem computed tomography (CT) scanning before opening of the vertebral canal to illustrate the vertebral and soft tissue lesions. CT images were obtained using a single slice helical CT machine (Emotion, Siemens, Erlangen, Germany). Slice thickness was 3 mm and images were reconstructed using filters for bone (spatial) and soft tissue (contrast) detail.

For histopathology, specimens of the soft tissue masses, perirenal fat, vertebrae, spinal cord and internal organs of case 1 were fixed in 10 % neutral buffered formalin. The tissue samples were processed routinely after decalcification of osseous tissues in a solution of 3.3 % formaldehyde and 17 % formic acid, sectioned at 4 µm and stained with haematoxylin and eosin. Selected sections were also stained using Masson’s trichrome method for connective tissue.

**Fig. 1:**
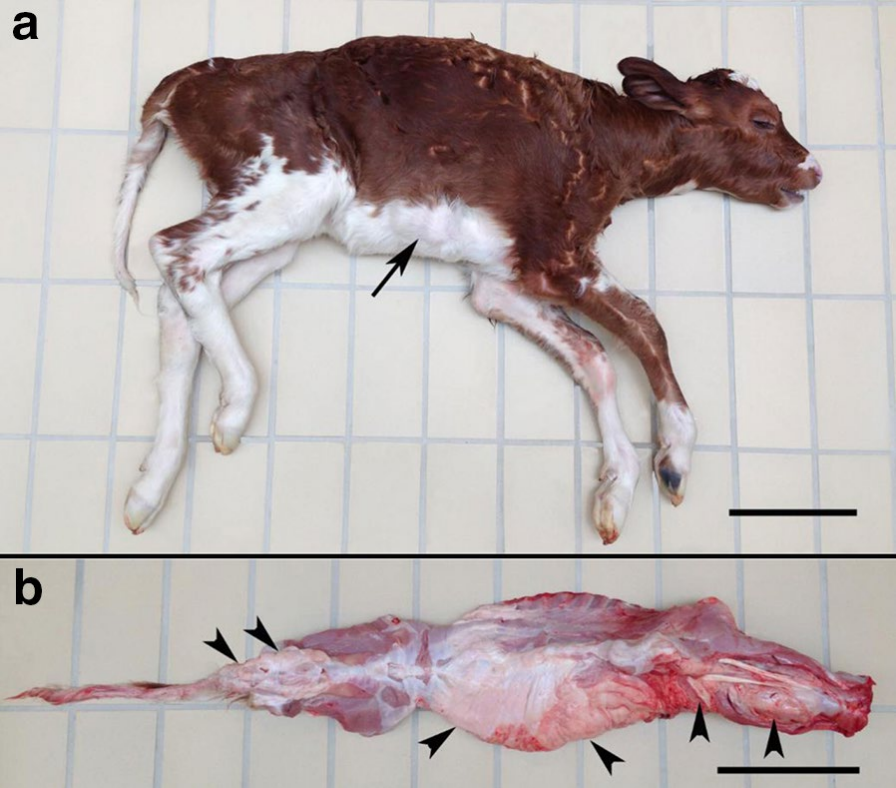
Gross lesions due to congenital infiltrative lipomas. **a** Notice the enlargement of the right side of the thorax due to large tumour masses. Local bone proliferation in the distal part of a rib is indicated by an *arrow*. **b** The widespread occurrence of the lipomas is evident after skinning (*arrow heads*). Dorsal view of the vertebral column. **a** and **b** *Bar* = 20 cm

**Fig. 2:**
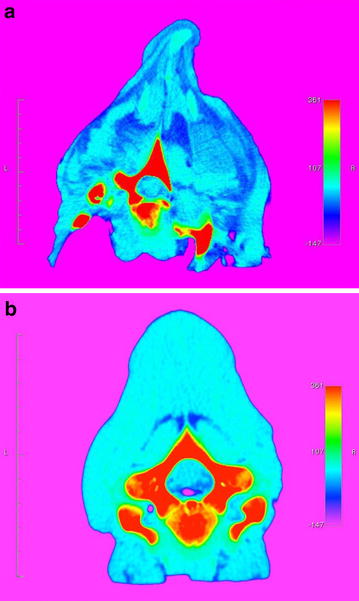
Post mortem computed tomography (CT) scanning images. **a** The diffuse infiltrative nature of the lipomas is seen as widespread *dark blue* coloring of the soft tissues. The vertebra and proximal parts of the ribs (*red color*) are distorted. **b** Comparative CT scanning of a normal neonatal Jersey calf at the same level as in **a** to illustrate the normal *lighter blue color* of muscles and normally developed bones. CT scanning at the level of the first thoracic vertebra; *scale* Hounsfield units

Skeletal malformations were externally visible in calf 1 as a 10 × 8 cm osseous swelling near the costa-sternal junction in the caudal right thorax and as increased length and slight lateral deviation of the thoracic spinous processes. Subcutaneous solid soft tissue swellings were palpated in the external thoracic wall and after skinning, widely distributed massive white, firm, soft tissue masses were found along the spine and covered almost the right external thorax and the dorsal parts of the left external thorax (Fig. 1). The masses compressed the surrounding muscles and bones and expanded by dissecting between muscles following intermuscular connective tissue and fascia. Diffusely distributed throughout the thoracic area were similar individual circumscribed masses up to 10 cm in diameter. Invasion of skin, bone or the parietal pleura was not found on gross examination. The infiltrative nature of the masses was seen at CT scanning as large soft tissue areas with CT numbers of −10 to −100 Hounsfield Units. These values are typical for voxels containing both adipose tissue and other tissues in various proportions (Fig. 2).

**Fig. 3:**
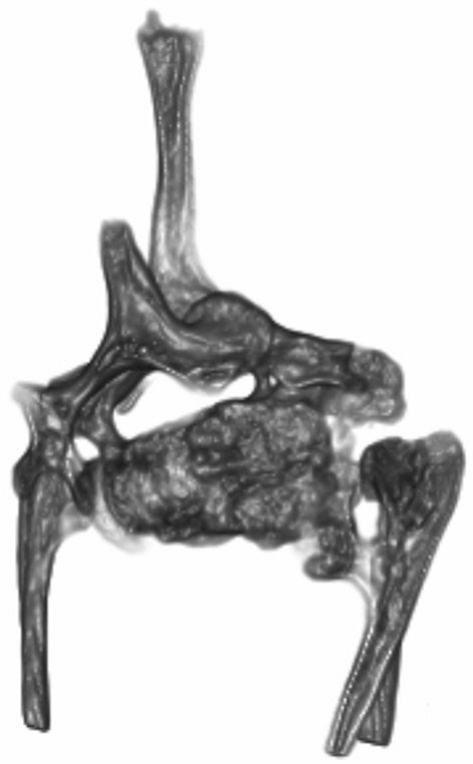
Surface rendered post mortem computed tomography (CT) scanning image. Notice the distorted morphology of the vertebrae, the abnormally shaped vertebral canal and the dislocation of the proximal part of the right rib. Specimen of the first two thoracic vertebrae

The caudal cervical and many of the thoracic vertebrae were malformed. The firm soft tissue masses intimately invaded spaces in and between vertebrae thus distorting and separating the vertebrae (Fig. 3). Soft tissue masses had invaded the vertebral canal of the caudal cervical vertebrae. The vertebral canal was distended and abnormally shaped in this region. The spinal cord was not compressed and could easily be separated from the tissue masses.

**Fig. 4:**
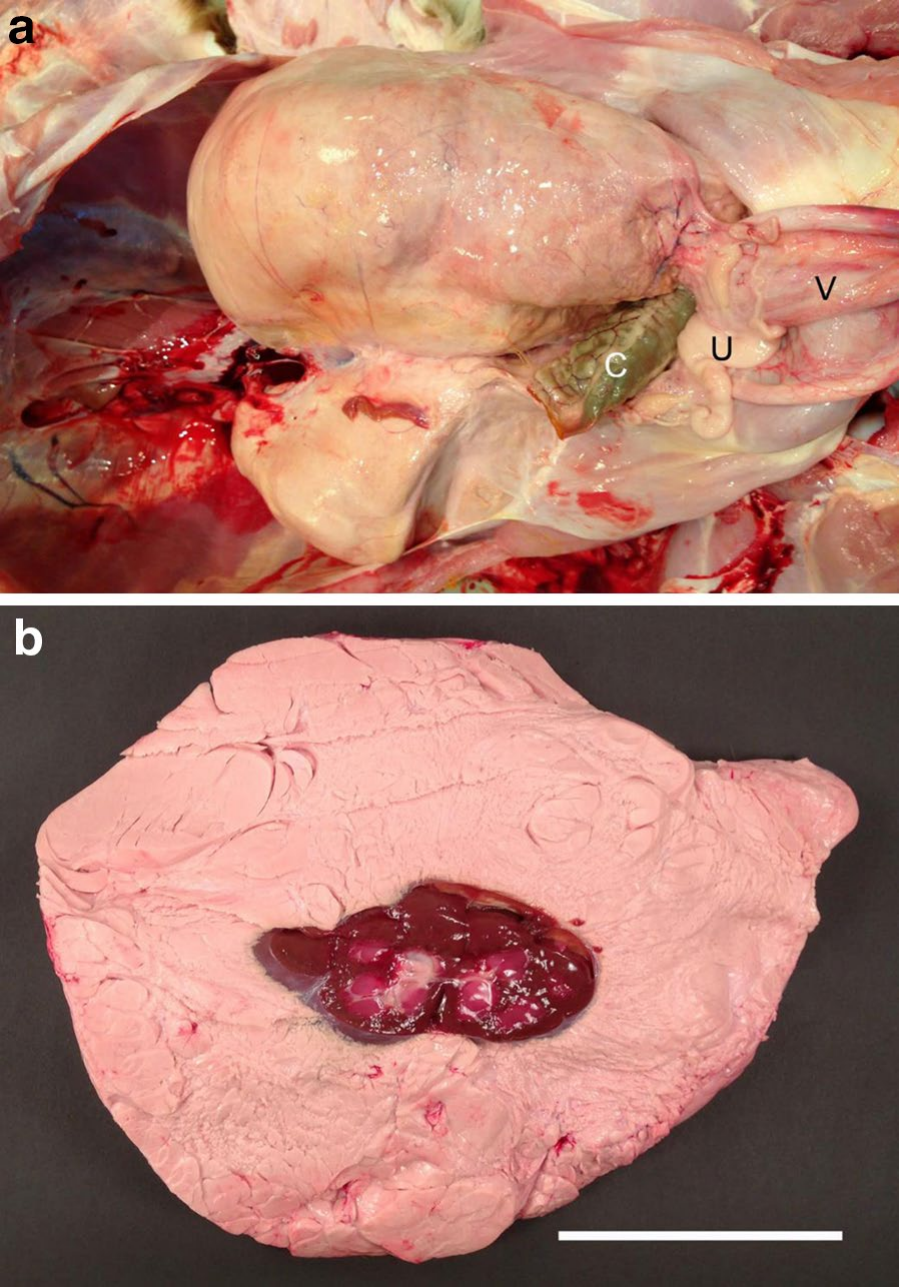
Bilateral retroperitoneal perirenal lipomas. **a** Abdominal lesions after partly evisceration. Notice the huge lipomas enclosing the kidneys, in particularly around the left kidney. *C* colon, *U* uterus and *V* vesica urinaria. **b** Longitudinal sectioning through the left kidney and the surrounding lipoma. *Bar* = 10 cm

Lesions in the abdomen were characterized by the presence of very large amounts of perirenal fat, especially around the left kidney where the mass measured 23 × 11 cm (Fig. 4a). The tissue morphology was similar to that of normal perirenal fat except for the increased amount of fat. The perirenal fat had a smooth peritoneal covering and was easily separated from the surrounding tissues and the kidneys. The intraabdominal masses caused the descending colon to be moved to the right. Normally developed kidneys and adrenal glands were found within the fat (Fig. 4b). The liver was slightly compressed. Other abdominal and all thoracic organs were normally developed as was the brain and spinal cord.

**Fig. 5:**
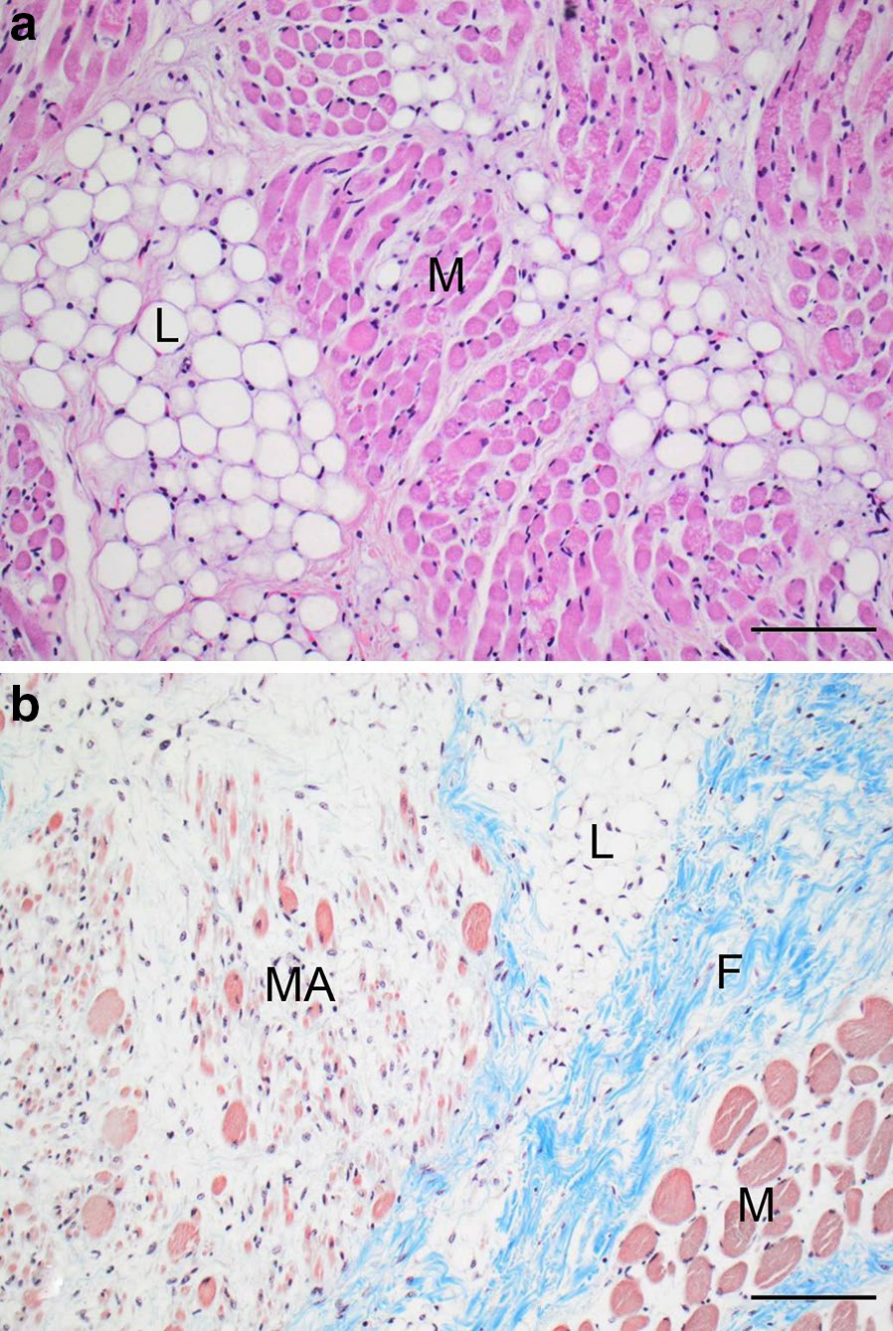
Photomicrographs of the congenital infiltrative lipomas. **a** Mature, morphological normal lipocytes (*L*) are infiltrating the muscle tissue (*M*) along the connective tissue streaks (perimysium). Haematoxylin and eosin, *bar* = 100 µm. **b** The infiltrative growth of the lipoma (*L*) is associated with fibrosis (*F*) and severe muscular atrophy (*MA*), while other muscle fascicles (*M*) are less affected. Masson’s trichrome stain, *bar* = 200 µm

Histology of the soft tissue swellings revealed muscle tissue that was heavily infiltrated by a uniform population of single vacuolated mature lipocytes. The invading lipocytes followed the connective tissue streaks (perimysium) in many places but locally, the lipocytic infiltration was more aggressive and invaded the individual muscular fascicles (Fig. 5a). Significant fibrosis was seen in many areas. Muscular atrophy was severe and widespread and included entire muscle fascicles as well as scattered individual muscle fibers (Fig. 5b). Although the lipocytes extended to the periosteum there was no periosteal invasion. Locally, the thickness of cortical bone was increased and with a lamellar morphology, which was especially evident in the malformed rib (hyperostosis). The periosteum was thickened with fibrosis on the external aspect of the periosteum. The bone marrow was almost completely replaced by lipocytes in some coccygeal vertebrae. In the vertebral canal, the lipocytes were located in the epidural space, but did not invade the dura mater. Similarly, the skin overlying the lesions was not affected. Except for an aggressive infiltrative growth, signs of malignancy were not observed.

The perirenal mass consisted of a uniform population of mature lipocytes with a single large cytoplasmatic vacuole and with sparse connective tissue. Neither signs of invasive growth nor signs of malignancy were observed. Except for a diffuse non-suppurative interstitial pneumonia, lesions were not present in other tissues.

Calf 2 had a localized chronic fibrous omphalitis and an umbilical hernia, but no findings indicative of tumours. No lesions were observed at histology of the internal organs.

## Conclusions

The affected calf had two types of lipomas, i.e., simple retroperitoneal lipomas and widespread infiltrative lipomas. As both types are rare, it is tempting to speculate that they have a similar pathogenesis although they differed significantly in their ability to invade the surrounding tissues. Two other cases of congenital infiltrative lipoma have been reported in cattle [2, 3], but none of these had simple lipomas. A single case of retroperitoneal perirenal lipomas in a calf has been reported [6], but that calf did not have infiltrative lipomas, so an association between the conditions remains hypothetical until other cases are reported.

The present case had malformation of the vertebrae. The vertebral distortion probably had its origin in the infiltrative growth of the lipoma during foetal development. The tumour invaded the vertebral canal through the intervertebral spaces and vertebral foramina and tumour masses may have compressed and distorted the developing vertebrae as reported in humans [10]. It is well known that infiltrative lipomas (infiltrative lipomatosis) in humans are associated with malformation of adjacent bones [11, 12] as also reported in a case of bovine congenital infiltrative lipoma [2]. The osseous enlargement of the rib was probably due to a periosteal reaction (hyperostosis), which was also a part of the vertebral lesions. It is recognized that human parosteal lipomas and infiltrative lipomas developing in close proximity to bones may induce bony hypertrophy, although the exact mechanisms are unknown [10–14]. In the present case, lipomatous tumour masses also invaded the bone marrow in the coccygeal vertebrae and the haematopoietic bone marrow was replaced by lipocytes. The very expansive and destructive growth of the infiltrative lipomas with malformation of bone demonstrates the aggressive potential of this benign tumour in calves.

The retroperitoneal lipomas had a morphology similar to the lipoma in a Holstein calf reported by Ikede [6]. These are benign tumours, but due to their large size may compress adjacent organs, e.g., causing hydronephrosis. Retroperitoneal lipomas of considerably sizes (several kg) have been reported in children, where the main clinical sign has been an enlarged abdomen of long duration [15]. As in humans, perirenal lipomas should be considered in calves with distended abdomen, even in cases without other signs of tumours.

## Ethics approval

This study did not require official or institutional ethical approval. The animals were handled according to good ethical standards and Danish legislation.
